# A Z-score based method for comparing the relative sensitivity of behavioral and physiological metrics including cognitive performance, mood, and hormone levels

**DOI:** 10.1371/journal.pone.0220749

**Published:** 2019-08-15

**Authors:** John A. Caldwell, Philip J. Niro, Emily K. Farina, James P. McClung, Gregory R. Caron, Harris R. Lieberman

**Affiliations:** 1 Oak Ridge Institute for Science and Education, Belcamp, MD, United States of America; 2 Military Nutrition Division, U.S. Army Research Institute of Environmental Medicine, Natick, MA, United States of America; 3 Henry M. Jackson Foundation for the Advancement of Military Medicine, Bethesda, MD, United States of America; 4 SERE EAST, Center for Security Forces, Brunswick, ME, United States of America; Auburn University - Harrison School of Pharmacy, UNITED STATES

## Abstract

A method for assessing the relative sensitivity of research metrics is proposed and illustrated by comparing 18 outcome measures from a published study of the cognitive, mood, and hormonal effects of four different levels of stress induced by intense military training. Research on the human response to stress often assesses multiple disparate dependent measures. Selecting the most sensitive is difficult as formal methods to compare varied dependent measures have not been developed. The method first converts the outcome measures into standard scores (z‐scores) and then compares them using analysis of variance to determine whether there are differences in how they assess the impact of graded levels of exposure to stress. The analysis detected various significant interactions in several measures and suggests self‐report mood questionnaires were more sensitive to the stressors present in the study than the cognitive or hormonal measures which were used. These findings support the effectiveness of the z‐score based method as a useful procedure for objectively evaluating the differential sensitivity of various metrics. This method could be useful for research on other independent variables when use of multiple assessment strategies is appropriate. It could be used for evaluating studies yielding conflicting results, such as those detecting effects on one parameter but not others. In such instances, cross‐metric inconsistencies may be due to differential sensitivity of measurement strategies rather than actual differences in the effects of the independent‐variable on the domains under investigation.

## Introduction

A major challenge of conducting research intended to improve the performance of many occupations, sports, and military activities—where optimal physical and cognitive function is critical for success, safety, and productivity—is selection of metrics that are both relevant to the independent variables of interest and sensitive to the impact these variables may exert upon performance, mood, and physiology. Unfortunately, there is a lack of consensus on which metrics are most sensitive, reliable, and valid for assessing human behavior [1]. This is especially true for studies that examine the wide range of physical, biochemical, and psychological domains that underlie human performance in real-world settings. Although research studies often assess numerous dependent measures and collect data so that statistical hypotheses testing can be conducted (occasionally accompanied by between-metric correlational analyses), the relative sensitivity of the various measures is rarely assessed.

In this paper, we propose the use of standardized scoring (z-score conversions) for assessing the relative sensitivity of different dependent measures. Standardizing the outcome data places all of the results from the various tests used in an investigation on the same comparable scale. Although this is not a routine practice in studies assessing multiple metrics (each of which may have different means, standard deviations, and ranges), if used it could be facilitate comparison of effect magnitudes and thus contribute to evaluation of test sensitivity. The normalization of data either is already in use or has been proposed in divergent areas such as prenatal screening of fetal biometric data [2], research in educational management [3], assays for external quality determinations in medical laboratories [4], neurocognitive test outcomes for longitudinal tracking of disease-related cognitive impairments [5], high-throughput image-based cell profiling [6], and others. In addition, the World Health Organization has recommended the standard scoring technique (z-scoring) to facilitate data-quality assessments of anthropometric data [7].

In the present manuscript we illustrate the utility of a new data normalization procedure using data from a previously published study of intense military stress [8]. We have chosen to use data from a “stress study” since the optimal approach to evaluate the effects of physical, psychological, or biological stressors has long been an area of contention. In particular, the relative sensitivity of self-report/subjective questionnaires versus more objective tests of cognitive performance and/or objective biochemical tests has been a matter of some debate in this arena.

## Methods

Data from a previously published Survive, Evade, Resist and Escape (SERE) school investigation of four graded levels of stress provides an appropriate model to examine the sensitivity of different types of tests. The lowest level of stress was the initial baseline test session conducted during classroom training; and the two highest levels of stress were associated with two different, very intense scenarios conducted during a mock prisoner of war (POW) captivity simulation. A moderate level of stress was present during a final test session (conducted 12 h after the second POW captivity) when volunteers remained in the POW scenario but were not exposed to an intense training scenario. Additional details of the SERE study are provided below along with the procedure for comparing the relative sensitivity of the tests used in that study.

### Materials

Thirty-four of the Navy and Marine uniformed personnel (see Table 1) who served as volunteers had complete data sets for the 3 cognitive/mood tests and the hormonal data of interest. The dependent measures of interest were from: 1) the Psychomotor Vigilance Task (PVT)—a sustained attention test in which subjects were required to rapidly respond to numerous visual stimuli presented at random intervals; 2) the Profile of Mood States (POMS)—a standardized self-report mood inventory in which subjects rated their current feelings as described by 65 mood adjectives, which yield sub-scale scores for Tension/Anxiety, Depression/Dejection, Anger/Hostility, Vigor/Activity, Fatigue/Inertia, Confusion/Bewilderment, and Total Mood Disturbance); and 3) the Match-to-Sample test—an assessment of short-term spatial memory in which subjects determined whether or not two matrix patterns presented in succession were different or identical after a brief delay (8 or 16 seconds). In addition, biochemical measures of stress were included as a fourth category–saliva assays of cortisol, testosterone, brain-derived neurotrophic factor (BNDF), and neuropeptide Y (NPY)–metabolites known to be associated with the stress response. Each of these assessments produced several outcome metrics from which a total of 18 were selected for inclusion in the sensitivity analysis (see Table 2). For clarity purposes, each of the individual variables was labeled as a “Dependent Measure,” and for reference, original untransformed data (baseline means and standard deviations) for each measure are presented in Table 3.

**Table 1:** Participant characteristics.

Demographic	N	Percent
**Gender**		
Male	31	91.2
Female	3	8.8
	**Mean**	**SD**
**Age**	25	3.5
**Height (in)**	69.8	2.8
**Weight (lbs)**	177.2	23.0
**BMI (kg/m**^**2**^**)**	25.2	2.4

**Table 2:** Detailed listing of the dependent measures examined.

Measure Number	Test	Dependent Measure
1	Psychomotor Vigilance Test (PVT)	Number Premature Responses
2	Psychomotor Vigilance Test (PVT)	Number TimeOut Errors
3	Psychomotor Vigilance Test (PVT)	Number Correct Hits
4	Psychomotor Vigilance Test (PVT)	Mean Reaction Time
5	Profile of Mood States (POMS)	Total Mood Disturbance
6	Profile of Mood States (POMS)	Tension-Anxiety
7	Profile of Mood States (POMS)	Depression-Dejection
8	Profile of Mood States (POMS)	Anger-Hostility
9	Profile of Mood States (POMS)	Vigor-Activity
10	Profile of Mood States (POMS)	Fatigue-Inertia
11	Profile of Mood States (POMS)	Confusion-Bewilderment
12	Match-to-Sample 8&16-sec Delays Combined	Number Correct Matches
13	Match-to-Sample 8&16-sec Delays Combined	Number TimeOut Errors
14	Match-to-Sample 8&16-sec Delays Combined	Mean Reaction Time
15	Hormone 1	Cortisol
16	Hormone 2	Testosterone
17	Hormone 3	BDNF
18	Hormone 4	NPY

**Table 3:** Baseline means and standard deviations of each dependent measure.

Dependent Measure	Mean	Standard Deviation
**Baseline PVT**		
Premature Response	10.7	9.5
TimeOut Errors	11.5	20.6
Number Correct	114.6	22.4
Reaction Time (sec)	0.30	0.03
**Baseline POMS**		
Total Mood Disturbance	20.0	17.8
Tension-Anxiety	6.6	3.8
Depression-Dejection	3.7	4.4
Anger-Hostility	7.2	6.3
Vigor-Activity	10.7	5.7
Fatigue-Inertia	7.1	5.0
Confusion-Bewilderment	6.2	3.1
**Baseline Match-to-Sample**		
Number Correct	15.4	3.4
TimeOut Errors	0.09	0.3
Reaction Time (sec)	4.5	1.6
**Hormones**				
Cortisol (μg/dL)	0.2	0.1
Testosterone (pg/mL)	56.7	25.0
BDNF (pg/mL)	11.6	24.8
NPY(pmol/L)	84.5	29.4

### Procedures to compare metrics

**Fig 1:**
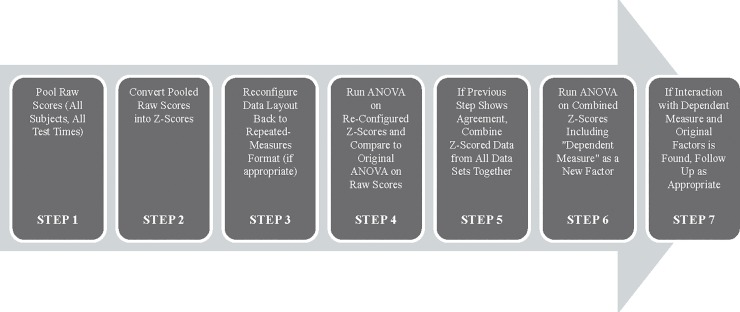
Summary of the data transformation and analysis procedure.

Multiple steps were required to prepare the data for conversion to ensure the conversion was performed correctly and for analyzing the data. This procedure is outlined in Fig 1. First, the repeated-measures nature of the data was removed so that all subjects and sessions for each dependent-measure could be pooled to allow calculation of the grand $\bar{X}$ and σ. Next, all data from each dependent-measure were converted to standard scores by $z=(X-\bar{X})/\sigma$ [9]. Next, for quality-control, the repeated-measures format was re-applied to the standardized data so that one-way ANOVAs could be performed on each metric and compared to the ANOVA originally performed on the untransformed data. The purpose of this was to ensure the F and p values were identical in both sets of ANOVAs, and that the $\bar{X}$ and σ of the z-scores for each dependent measure equaled “0” and “1” respectively (as expected). Afterward, the z-scores for all 18 of the dependent-measure data sets were aggregated into a single input file and then analyzed via two-way ANOVA for Time (test sessions 1–4) and Dependent Measure (test metrics 1–18)—(a 4 x 18 ANOVA). The results were then examined to determine whether there was a time-by-measure interaction to determine if one or more of the assessments (dependent measures) was differentially affected by the stressors of SERE school (i.e. that there was a difference across the four time points on some measures, but not on others or that the pattern of differences on some measures was not consistent with the pattern of differences on others). If statistically significant effects were observed, appropriate pairwise comparisons and polynomial contrasts were conducted. Since the purpose of the method was to compare the sensitivity across measures rather than determining whether the stressors of SERE school affected cognition, mood, and physiology, no protection against alpha inflation was applied.

## Results

It was predicted the two-way interaction from the overall ANOVA would be significant since analyses of the untransformed raw-score data sets had previously indicated some of the dependent measures were affected by SERE school stress, whereas others were not. Also, it was predicted that the main effect of time (testing session) would be significant since the low-stress baseline and moderate stress recovery test sessions from SERE school were being compared to the two high-stress training sessions. It was not, however, expected that the main effect for “dependent-measure” would be significant since once the data were transformed into z-scores, the grand mean for each z-scored data set would equal “0”. The ANOVA revealed that the expected interaction and main effects did in fact occur since the main effect of time was significant (F(2.9,96.5) = 31.66, p < .0001), the dependent-measure main effect was not significant (F(5.8,191.8) = .001, p = 1.000), and the time-by-measure interaction was significant (F(11.7,263.5) = 9.85, p < .0001).

**Fig 2:**
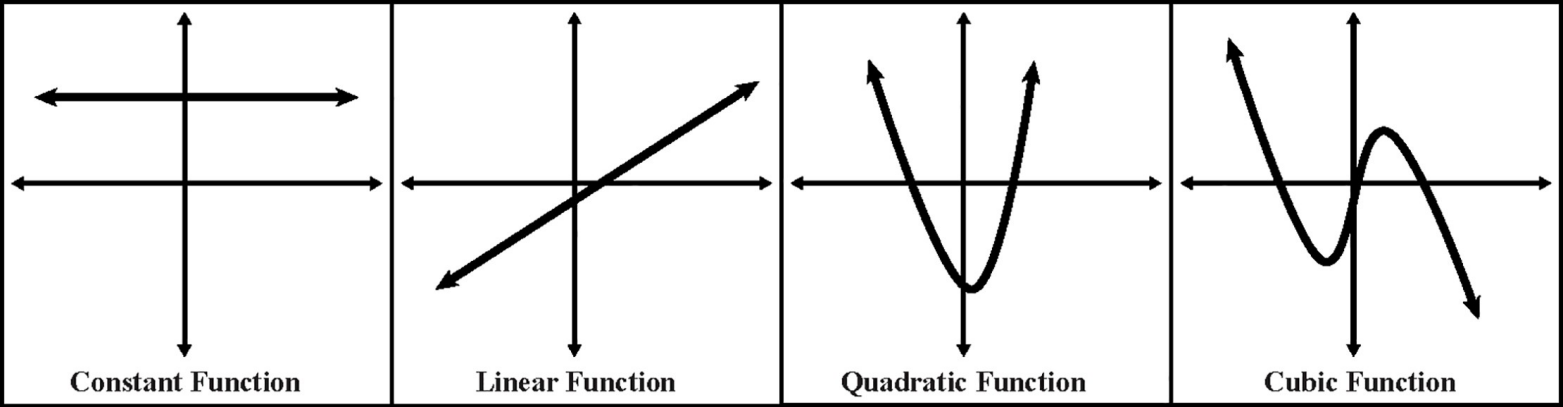
Function for which each orthogonal trend-analysis contrast was tested for significance p < .05.

**Fig 3:**
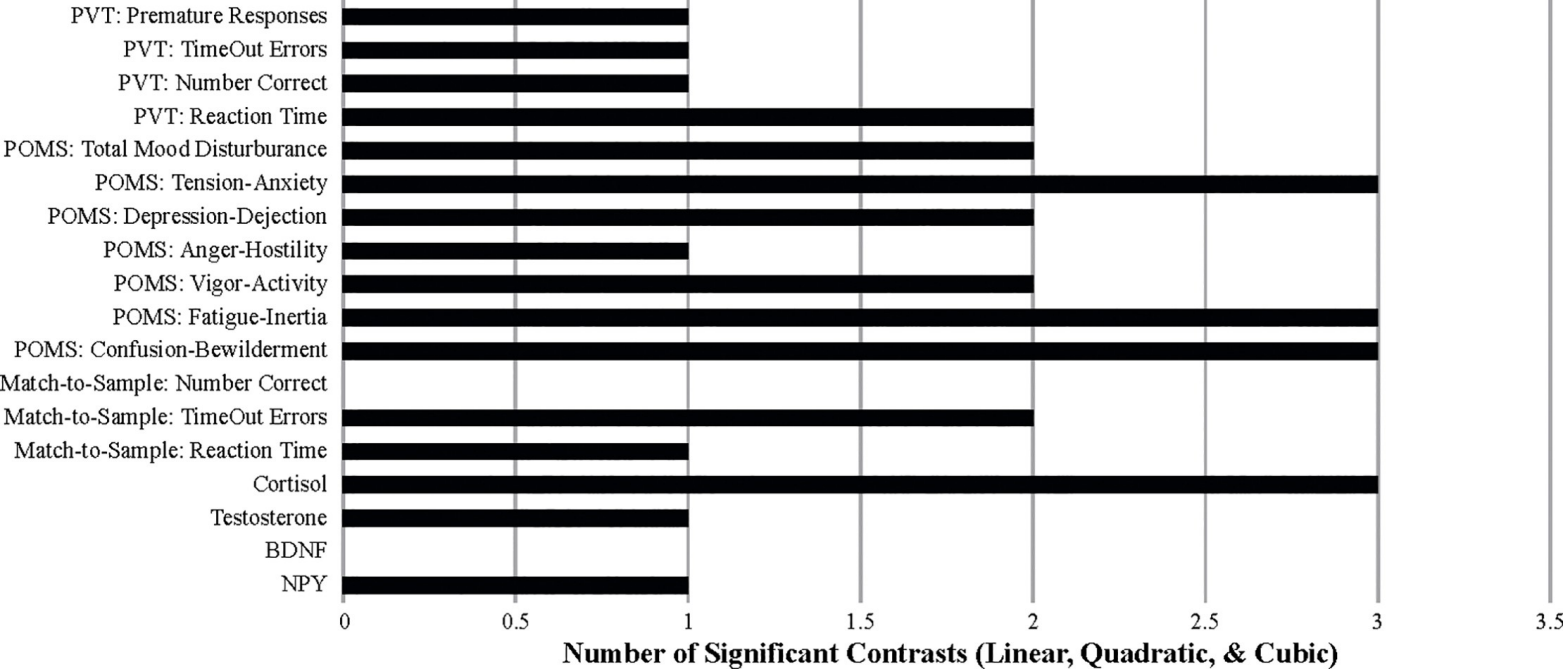
The number of trend-analysis contrasts that were significant p < .05.

**Fig 4:**
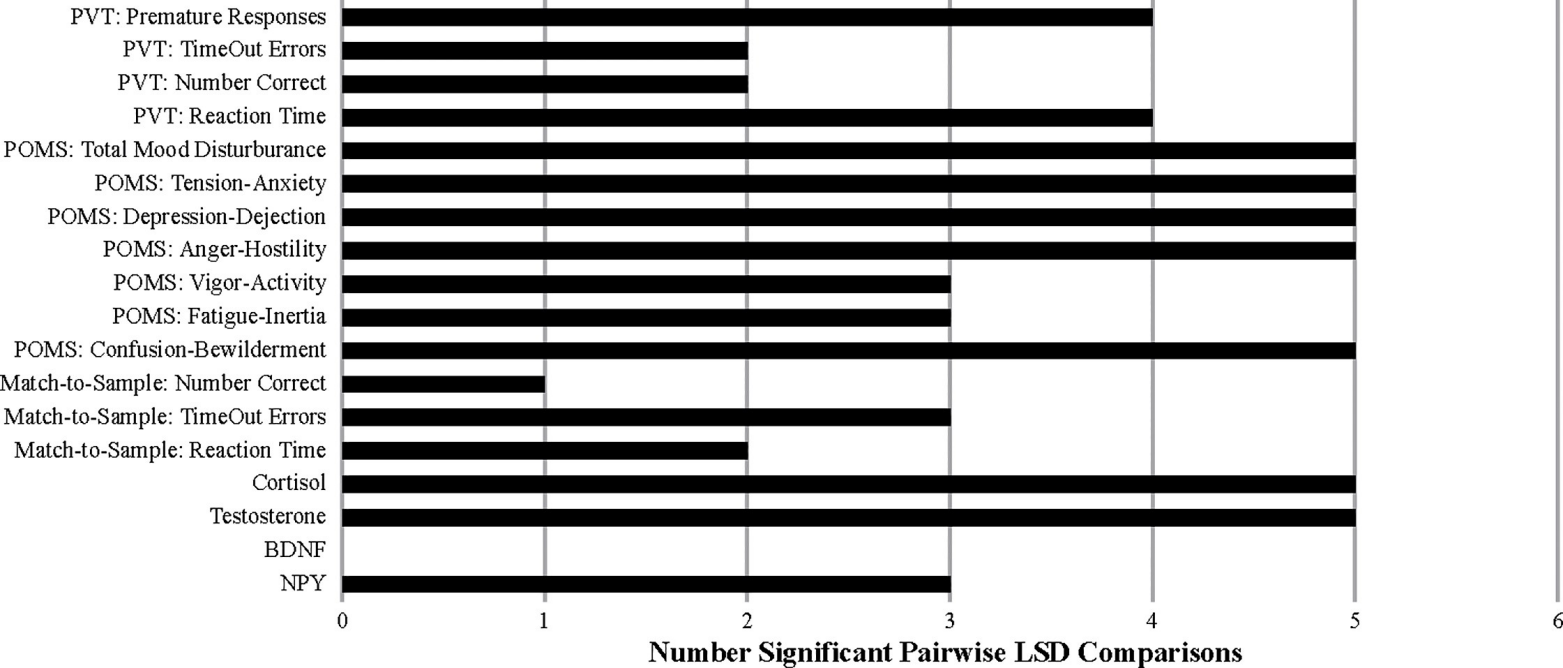
The number of pairwise post-hoc comparisons that were significant p < .05.

To follow up on the above finding of “overall significance,” the significant time-by-measure interaction was examined using one-way ANOVAs on each dependent-measure data set individually to identify any that were not significant across the 4 testing times since this would indicate the measure in question was not sensitive to the stresses of SERE school. In addition, orthogonal polynomial contrasts [9] across the 4 testing times of all dependent measures were conducted to determine whether linear, quadratic, or cubic trends were present, focusing primarily on the quadratic since this was the expected pattern of interest (since it was predicted that there would be a significant change from the baseline session to the first and second high-stress situations followed by a return or near-return to baseline during the recovery session). The orthogonal trends that were computationally possible given the number of data points over time (4) are provided in Fig 2. For those measures showing a significant effect of time (from the one-way ANOVAs), Fisher’s Least Significant Difference (LSD) post-hoc pairwise comparisons were performed. The outcome of these post-hoc examinations are displayed in Table 4 and measures which resulted in significant individual ANOVA results are shaded in light grey. The number of significant contrasts and pairwise comparisons (regardless of whether the individual ANOVAs revealed a time main effect) are graphically depicted in Figs 3 and 4.

**Table 4:** Summarized results from the follow-up statistics for each dependent measure.

Assessment Variable	Sig. One-Way?	Sig. Linear?	Sig. Quad-ratic?	Sig. Cubic?	Time 1 vs Time 2	Time 1 vs Time 3	Time 1 vs Time 4	Time 2 vs Time 3	Time 2 vs Time 4	Time 3 vs Time 4	No. Sig. Cont-rasts	No. Sig. Pair-wise
**PVT**												
Premature Response	No/ p = .059	No/ p = .788	No/ p = .179	Yes/ p = .033	.050	0.548	.036	.045	0.153	.043	1	4
TimeOut Errors	No/ p = .108	No/ p = .369	No/ p = .301	Yes/ p = .005	0.771	.091	0.750	.002	0.318	.0003	1	2
Number Correct	No/ p = .061	No/ p = .500	No/ p = .324	Yes/ p = .001	0.517	.090	0.971	.001	0.341	.0001	1	2
Reaction Time	Yes/ p = < .0001	Yes/ p = .001	No/ p = .070	Yes/ p = .002	.0003	.007	.0001	0.106	0.892	.051	2	4
**POMS**												
Total Mood Disturbance	Yes/ p = < .0001	Yes/ p = < .0001	Yes/ p = < .0001	No/ p = .122	< .0001	< .0001	< .0001	0.442	.001	.0001	2	5
Tension-Anxiety	Yes/ p = < .0001	Yes/ p = < .0001	Yes/ p = < .0001	Yes/ p = .045	< .0001	< .0001	< .0001	0.459	.0003	.0003	3	5
Depression-Dejection	Yes/ p = < .0001	Yes/ p = .013	Yes/ p = < .0001	No/ p = .721	< .0001	< .0001	.005	0.547	.0003	.0002	2	5
Anger-Hostility	Yes/ p = < .0001	No/ p = .104	Yes/ p = < .0001	No/ p = .435	< .0001	.0002	.0115	0.239	.015	.0002	1	5
Vigor-Activity	Yes/ p = < .0001	Yes/ p = < .0001	Yes/ p = .001	No/ p = .071	< .0001	< .0001	< .0001	0.695	0.626	0.421	2	3
Fatigue-Inertia	Yes/ p = < .0001	Yes/ p = < .0001	Yes/ p = < .0001	Yes/ p = .001	< .0001	< .0001	< .0001	0.862	0.114	0.119	3	3
Confusion-Bewilderment	Yes/ p = < .0001	Yes/ p = .001	Yes/ p = < .0001	Yes/ p = .043	< .0001	< .0001	< .0001	0.626	.001	.001	3	5
**Match-to-Sample**												
Number Correct	No/ p = .122	No/ p = .077	No/ p = .100	No/ p = .736	.094	.040	.080	0.806	1 < .0001	0.767	0	1
TimeOut Errors	Yes/ p = .003	Yes/ p = .025	Yes/ p = .005	No/ p = .269	0.571	0.661	.017	1 < .0001	.003	.003	2	3
Reaction Time	Yes/ p = .043	No/ p = .146	No/ p = .280	Yes/ p = .002	.016	0.267	.057	.018	0.635	0.255	1	2
**Hormones**												
Cortisol	Yes/ p = < .0001	Yes/ p = < .0001	Yes/ p = < .0001	Yes/ p = .001	.003	< .0001	< .0001	< .0001	0.104	.001	3	5
Testosterone	Yes/ p = < .0001	No/ p = .625	No/ p = .312	Yes/ p = < .0001	< .0001	0.226	.021	< .0001	.001	< .0001	1	5
BDNF	No/ p = .499	No/ p = .346	No /p = .464	No/ p = .419	0.458	0.659	0.140	0.524	0.642	0.255	0	0
NPY	Yes/ p = .005	Yes/ p = .002	No/ p = .527	No/ p = .228	0.232	0.123	.003	0.920	.014	.014	1	3

**Fig 5:**
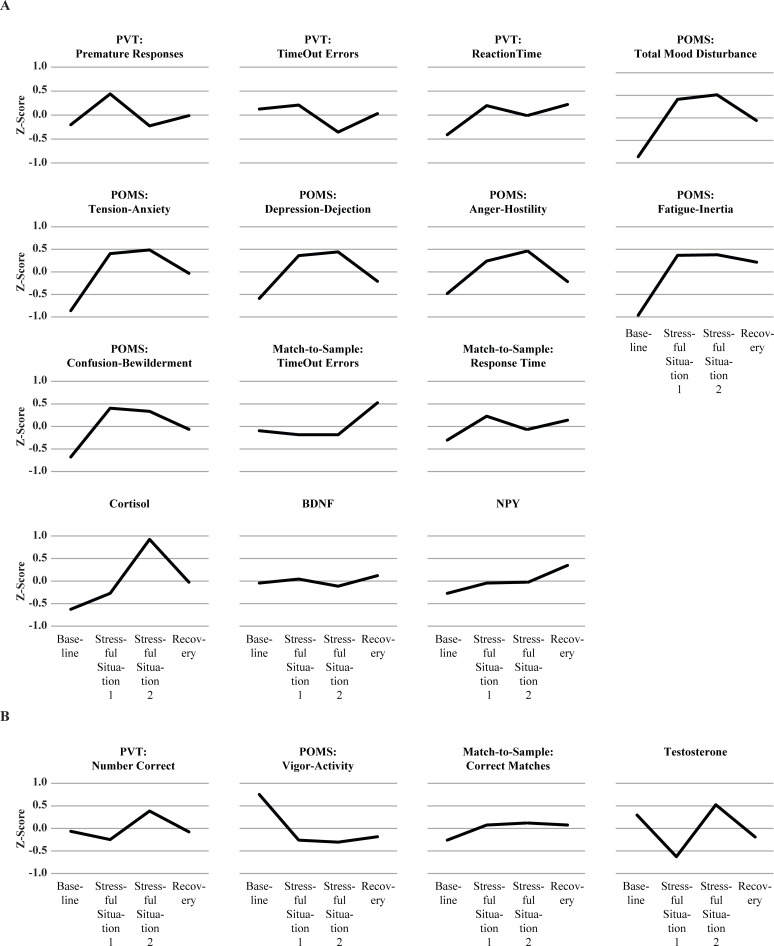
Measures expected to have positive (5A) and negative (5B) trends from baseline to captivity.

After all analyses were complete, the z-score means for each individual Dependent-Measure data sets at baseline (Time 1), stressful situation 1 (Time 2), stressful situation 2 (Time 3), and recovery (Time 4) were graphically depicted using a standardized scale which ranged from +1.0 to -1.0 to further characterize the pattern of results. To aid in visual comparisons among the different measures, these graphs are arranged so that measures anticipated to change in a positive direction from baseline (increase due to stress) to captivity are grouped together (Fig 5A). Measures that were expected to change in a negative direction from baseline (decrease due to stress) to captivity are grouped and presented in Fig 5B.

**Fig 6:**
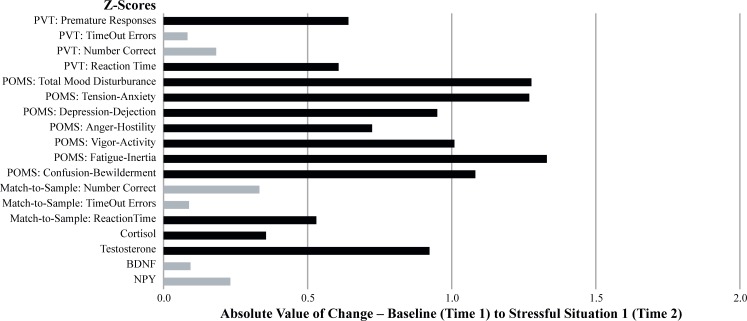
The magnitude of the mean change between test sessions 1 and 2.

**Fig 7:**
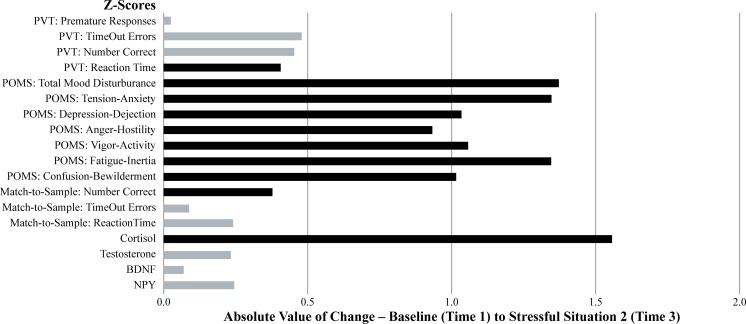
The magnitude of the mean change between test sessions 1 and 3.

To further illustrate the sensitivity of each dependent measure, the absolute value of the change from baseline (Time 1) to stressful situation 1 (Time 2), in terms of z-scores, was calculated and graphed (see Fig 6). As an indication of which measure revealed a significant pairwise change from baseline (Time 1) to stressful situation 1 (Time 2), those that were p < .05 are depicted in dark grey and those that were p>.05 are depicted in light grey. The same procedure was followed for the absolute change from baseline (Time 1) to stressful situation 2 (Time 3) and are illustrated in Fig 7. Additional comparisons between baseline (Time 1) and recovery (Time 4) were not conducted because it was expected that most metrics would indicate there was a return towards baseline values during the recovery period, and thus, a comparison between baseline and recovery would not provide any additional information regarding the relative sensitivity of the various assessments.

The graphical and tabular data considered together indicate the most sensitive measures of the stress associated with SERE school were the self-reported mood variables. There were significant overall time effects, significant quadratic trends, significant baseline vs. stressful training situation 1 (Time 1 vs. Time 2) pairwise comparisons, and significant baseline vs. stressful training situation 2 (Time 1 vs. Time 3) pairwise comparisons on all of the POMS subscales—Total Mood Disturbance, Tension/Anxiety, Depression/Dejection, Anger/Hostility, Fatigue/Inertia, Confusion/Bewilderment, and Vigor/Activity. Testosterone and NPY as well as the cognitive measures associated with response timing were next in terms of sensitivity as demonstrated by the overall significant time effect on both of these hormone levels, as were PVT Reaction Time, Match-to-Sample Reaction Time, and Match-to-Sample Time Outs. The follow-up statistical tests (i.e., both the trend-analysis contrasts and the pairwise comparisons) on these measures were not as definitive as POMS and cortisol measures since only 1 of the 6 assessments (Match-to-Sample Time Outs) revealed a significant quadratic trend, and only 3 of the 6 (PVT Premature Responses, Match-to-Sample Number Correct, and Match-to-Sample Reaction Time) showed significant baseline vs. stressful situation 1 (Time 1 vs. Time 2) pairwise contrasts. Only 1 of the 6 (PVT Reaction Time) changed significantly from baseline (Time1) to stressful situation 2 (Time 3). None of the remaining assessments (BDNF or the cognitive measures associated with accuracy—Number Correct) were affected by the stress of SERE school.

## Discussion

The primary objective of this study was to describe and evaluate the utility of a method for determining the differential sensitivity of various behavioral and physiological metrics. As a test case, the method was applied to data from an investigation that assessed the impact of exposure to intense stress induced by SERE school, a well-documented, high stress, multi-stressor environment with a known pattern of effects over time [8, 10, 11–13].

The z-score derived method described here indicated that measures obtained with a standardized and validated mood scale better characterized the impact of the multi-stressor environment than the cognitive tests and hormone assays used in the study. This conclusion is based on several observations. First, overall analysis of the 18 metrics tested here revealed substantial differences in sensitivity to the impact of intense stress as indicated by a significant time-by-measure interaction on overall ANOVA. Second, the majority of post-hoc examinations of the standardized POMS measures (i.e., significance on the trend-analysis contrasts and the post-hoc pairwise comparisons) were significant, but this was not the case for the other measures with the exception of cortisol. Only one other measure showed a significant stress-related trend, and only half had significant pairwise comparisons when the non-stressful baseline was compared to the two highly-stressful training sessions. This was not the case with the POMS measures where the number of significant trends was greater and most of the pairwise comparisons were significant. Third, the graphically-displayed pattern of effects on nearly every POMS subscale followed the expected “inverted U-shape” function from baseline to stressful training situations 1 and 2, and then the recovery period. This pattern generally was not apparent in the data from the other behavioral and physiological measures. Finally, and most important, the magnitude of changes from baseline to both of the stressful training points for the various POMS subscales exceeded the values of the other dependent measures–placing all measures on the same scale via data standardization (via z-scores) was critical for making this comparison.

The overall results of the present analysis were consistent with the findings from several other previously published studies on various types of stress. For example, Saw, et al. [14] reported mood scales and well-being questionnaires were more sensitive to the effects of acute and sustained athletic training than measures such as blood markers, heart rate, and oxygen consumption. In addition, Verde et al [15] observed mood changes provided a better indication of overstress in athletes than resting heart rate, perceived exertion during submaximal running, sleep quality, and/or orthopedic injuries. Our results also are consistent with those of Caldwell et al. [16] who, in a study of fighter pilots, demonstrated substantially greater sleep-deprivation-related changes in POMS measures than on cognitive performance measures such as mathematical processing and psychomotor tracking. In that study, self-reported fatigue and alertness ratings also were better at predicting operationally-relevant performance (flight performance) than measures of eye-movement saccadic velocity, EEG activity, psychomotor tracking, and mathematical processing [17]. These findings are also in agreement with an earlier study of Johnson and Naitoh [18] which found that self-reported fatigue ratings in response to sleep loss were greater than decrements in cognitive performance. Finally, the results from the present study are consistent with investigations of other militarily-relevant stressors such as severe undernutrition [19], mild dehydration [20], and a multistressor field environment [21]. The results are also consistent with those of another SERE study [22].

## Conclusions

Overall, the z-score based methodology described here for standardizing and analyzing data from multiple types of dependent measures appears to provide an objective method to assess the differential sensitivity of such measures. Conducting such analyses could be useful for planning research on stress and other domains. Furthermore, such analyses could aid in the interpretation of conflicting results from a given study since differences across measures could reflect differences in sensitivity of the tests used. It appears that in some instances investigators conclude that different functions (e.g. one aspect of cognitive function such vigilance vs. another such as working memory) are differentially sensitive to a specific treatment when in fact the differences may actually be attributable to differences in test sensitivity not the underlying function.

Of course, test sensitivity is only one of the factors that must be considered in planning studies. Other factors such as discriminant and construct validity (the extent to which measures relate to one another and reflect the construct they are designed to reflect), reliability (the extent to which measures provide consistent results), specificity (the extent to which measures are unaffected by extraneous factors), generalizability (the degree to which measures reflect the same effect across all tested individuals), and practical feasibility must also be considered [23]. Nevertheless, test sensitivity is an important issue since human research is extremely resource intensive. Use of less than optimal tests or dependent measures can result in failure to reveal real overall treatment effects (Type II statistical errors), especially if those effects are relatively subtle. Choosing the most sensitive metrics will improve overall research efficiency as well as the applicability of the research. Thus, if appropriate, we suggest the addition of test-sensitivity analysis to the usual statistical assessments conducted.

## Limitations

We have described an objective approach to explore test sensitivity and suggest how it could be useful. The procedure described does not completely resolve the issue of the optimal tests to use in stress research (nor was it intended to), but rather provides a method for comparing such tests. It could be used with existing data sets or data from future studies to develop a body of literature that addresses the issue of test sensitivity. When interpreting the present findings, several limitations should be noted. First, we have illustrated the benefits of the technique on data from a single study in which the independent variable was known to produce powerful effects. Whether or not the procedure we propose would be as useful for studies of other domains or less severe stress exposure has not yet been determined. Cognitive tests or biochemical markers not included here could be more sensitive than self-reported mood questionnaires, and analysis of other types of stress could yield different findings. Second, the technique itself is time consuming (although it could be automated), and while it provides an objective measure of test sensitivity (i.e. from ANOVAs and post-hoc testing), a degree of judgment remains. The investigator must determine how much of a difference in the number and magnitude of statistically-significant results is needed to indicate a “practically-significant” difference in the sensitivity of the measures being compared. Also, it should be noted that adding the sensitivity analysis described here to a standard manuscript would add substantially to its length. Nevertheless, just as statistical power analyses have become a standard practice for determination of sample sizes and experimental procedures at the outset of research, it would often be useful to know the relative sensitivity of the metrics used in a completed study. Third, in the present investigation, where our aim was to compare the sensitivity of different test metrics rather than to determine the effects of SERE school on cognition and performance, we did not apply permutation testing to empirically decide whether the number and magnitude of the results occurred by chance or not. Permutation tests are becoming increasingly popular for the control of potential false positives [24] and should be considered for multivariate designs.
